# Genomic profile of human meningioma cell lines

**DOI:** 10.1371/journal.pone.0178322

**Published:** 2017-05-26

**Authors:** Yu Mei, Wenya Linda Bi, Noah F. Greenwald, Nathalie Y. Agar, Rameen Beroukhim, Gavin P. Dunn, Ian F. Dunn

**Affiliations:** 1 Center for Skull Base and Pituitary Surgery, Department of Neurosurgery, Brigham and Women's Hospital, Harvard Medical School, Boston, Massachusetts, United States of America; 2 Department of Cancer Biology, Dana-Farber Cancer Institute, Boston, Massachusetts, United States of America; 3 Broad Institute of MIT and Harvard, Cambridge, Massachusetts, United States of America; 4 Department of Medical Oncology, Dana-Farber Cancer Institute, Boston, Massachusetts, United States of America; 5 Department of Neurosurgery, Pathology, and Immunology, Center for Human Immunology and Immunotherapy Programs, Washington University School of Medicine, St. Louis, Missouri, United States of America; University of California Los Angeles, UNITED STATES

## Abstract

Meningiomas, derived from arachnoid cap cells, are the most common intracranial tumor. High-grade meningiomas, as well as those located at the skull base or near venous sinuses, frequently recur and are challenging to manage. Next-generation sequencing is identifying novel pharmacologic targets in meningiomas to complement surgery and radiation. However, due to the lack of *in vitro* models, the importance and implications of these genetic variants in meningioma pathogenesis and therapy remain unclear. We performed whole exome sequencing to assess single nucleotide variants and somatic copy number variants in four human meningioma cell lines, including two benign lines (HBL-52 and Ben-Men-1) and two malignant lines (IOMM-Lee and CH157-MN). The two malignant cell lines harbored an elevated rate of mutations and copy number alterations compared to the benign lines, consistent with the genetic profiles of high-grade meningiomas. In addition, these cell lines also harbored known meningioma driver mutations in neurofibromin 2 (*NF2)* and TNF receptor-associated factor 7 (*TRAF7)*. These findings demonstrate the relevance of meningioma cell lines as a model system, especially as tools to investigate the signaling pathways of, and subsequent resistance to, therapeutics currently in clinical trials.

## Introduction

Meningiomas, arising from the meninges surrounding the brain and spinal cord, account for a third of all primary brain tumors [1]. The World Health Organization (WHO) classifies meningiomas into three grades, with increasing grade corresponding to increasing proclivity for invasion and recurrence. Grade I meningiomas are frequently curable with surgery. However, a subset located at the skull base, adjacent to major venous sinuses, or insinuated around major neurovascular structures pose challenges to complete resection. Furthermore, meningiomas of higher grades frequently recur despite surgery and radiation, with no effective alternative pharmacologic treatments.

Recently, an increasing understanding of the genomic basis underlying meningiomas have opened potential therapeutic targets for recurrent or progressive tumors [2–6]. In addition to mutation or loss of *neurofibromin 2* (*NF2*), recurrent mutations in *v-akt murine thymoma viral oncogene homolog 1* (*AKT1)* and *v-akt murine thymoma viral oncogene homolog 3* (*AKT3)*, *phosphoinositide-3-kinase catalytic alpha polypeptide* (*PIK3CA)*, *TNF receptor-associated factor 7* (*TRAF7*), *smoothened* (*SMO)*, *krupplelike factor 4* (*KLF4)*, *SWI/SNF related*, *matrix associated*, *actin dependent regulator of chromatin*, *subfamily b*, *member 1* (*SMARCB1)*, *RNA polymerase II subunit A* (*POLR2A)*, *telomerase reverse transcriptase (TERT)* promoter, *BRCA1 Associated Protein 1* (*BAP1)*, and *homolog of suppressor of fused (SUFU)* have been identified within subsets of meningiomas [2–7]. Furthermore, the burden of chromosomal gains and losses, or copy number status, of atypical meningiomas has been associated with recurrence risk [8]. However, the impact of these mutations and chromosomal instability on tumor initiation and progression remain incompletely defined, in part due to a lack of effective *in vitro* models for meningioma.

Meningioma cell lines with canonical oncogenic mutations or genome disruption may serve as effective model systems to interrogate the biological consequences of initiating genomic alterations and their inhibition. Thus, we profiled the exomes of four common meningioma cell lines to determine the representation of characteristic meningioma genomic features suitable for targeted investigations.

## Methods

### Human meningioma cell line culture

We profiled four established human meningioma cell lines, including two benign lines (HBL-52 and Ben-Men-1) and 2 malignant lines (IOMM-Lee and CH157-MN). The two benign meningioma cell lines were purchased from CLS Cell Lines Service (Eppelheim, Germany) and DSMZ (Braunschweig, Germany) respectively. CH157-MN was courtesy of Dr. Yancey Gillespie (University of Alabama-Birmingham), and IOMM-Lee was courtesy of Dr. Randy Jensen (University of Utah). All cell lines, except HBL-52, were cultured in growth media including Dulbecco's Modified Eagle Medium (DMEM), 10% fetal bovine serum, 2mM L-Glutamine, 100 IU/mL of penicillin and 100 μg/mL of streptomycin (Life Technology, Grand Island, NY). HBL-52 was cultured in McCoy’s 5A medium (Fisher scientific, Pittsburgh, PA) supplemented with 2mM L-Glutamine, 10% fetal bovine serum, 100 IU/mL of penicillin and 100 μg/mL of streptomycin. Cultured cells were maintained at 37° in a 5% CO2 atmosphere.

### DNA isolation

Human meningioma cells were cultured in T25 flask until confluence, washed with PBS, trypsinized in 0.25% Trypsin/EDTA (Life Technology, Grand Island, NY), and suspended in PBS. DNA was isolated using the QIAamp DNA mini kit (Qiagen, Valencia, CA) according to the manufacturer’s protocol. The concentration of double-stranded DNA was quantified using an Epoch Spectrophotometer (BioTek, Winooski, VT).

### Next-generation sequencing

Whole exome sequencing (WES) was performed at the Broad Institute and the Center for Cancer Genome Discovery at the Dana-Farber Cancer Institute, as described previously [2, 9]. Briefly, to generate 250bp libraries, 250ng/ul of purified DNA was randomly fragmented by Covaris sonication (Covaris, Woburn MA), followed by purification using Agencourt AMPure XP beads (Beckman Coulter, Inc., Indianapolis, IN) and ligation to DNA barcoded adaptors (Illumina TruSeq, Illumina Inc., San Diego, CA). Exome hybrid capture was performed with Agilent Sure Select All Exon v2.0 hybrid capture kit (Agilent Technologies, Santa Clara, CA). The 250bp libraries were loaded for paired-end sequencing on Illumina GAIIx (Illumina Inc., San Diego, CA). Sample reads were de-multiplexed using Picard tools [10, 11] (http://picard.sourceforge.net), read pairs were aligned to the hg19 reference sequence (NCBI 37) (ftp://ftptrace.ncbi.nih.gov/1000genomes/ftp/technical/reference/) using the Burrows-Wheeler Aligner [12]. Bias in base quality score assignments due to flow cell, lane, dinucleotide context, and machine cycle were analyzed and recalibrated, and local realignment around insertions or deletions (indels) was obtained using the Genome Analysis Toolkit (GATK) [13, 14], for generation of a single BAM file for each cell line for analysis of copy number alterations, mutations, and large-scale structural rearrangements.

### Genomic analysis

Single nucleotide variants were called and post-filtered using MuTect v1.1.4 [15], which were then annotated to genes and compared to events in the Catalogue of Somatic Mutations in Cancer using Oncotator. Indel calling was achieved using the GATK Somatic Indel Detector tool. As described previously [10], somatic indels were called from locally realigned data based on the fraction of supporting reads at a given locus in the tumor cell BAM file. Variants were characterized as somatic using a CEPH cell line as the normal control. Variants were filtered against SNP database as well as 1000 Genomes [16]. The resulting variants were input into the variant effect predictor (VEP) (http://www.ensembl.org/info/docs/tools/vep/index.html) to predict the consequences of the variants on the protein sequences.

Somatic copy-number alterations (SCNAs) were called using RecapSeg, which performs local change-point analysis and subsequent merging of adjacent chromosomal segments with similar copy numbers. The resulting segments were annotated using Oncotator, then visualized using integrative genomics viewer (IGV, http://www.broadinstitute.org/igv/). The SCNAs across the entire genome were then analyzed by GISTIC 2.0 [17, 18].

Rearrangement detection was performed using dRanger and BreakPointer algorithms [10, 19], and visualized using the Circos program (http://mkweb.bcgsc.ca/circos) [20]. However, exome sequencing is limited in power to detect rearrangements and few were detected.

### Targeted sequencing

Variants identified by WES, as well as selected regions of previously published genes implicated in meningioma (*NF2*, *AKT1*, *TRAF7*, *SMO*, and *KLF4*), were analyzed by PCR and Sanger sequencing. Mutant sites from *NF2* (22), *TRAF7* (5), *AKT1* (1), *SMO* (2), *KLF4* (1), and *TERT* (2) were sequenced. DNA from the four meningioma cell lines and one normal human cell line (293T) were amplified using PCR primers designed to target identified mutations (**S1 Table**). PCR products were separated by 1% agarose gel, visualized under UV light, extracted using the Qiagen Gel Extraction Kit (Valencia, CA), and subject to Sanger sequencing (Macrogen, Boston, MA). Mutant variants identified by Sanger sequencing were verified by alignment of sequenced results with sequences of normal human cell line 293T and human genomic DNA reference (hg19). Furthermore, the *TERT* promoter region was focally sequenced across cell lines to assess for presence of *TERT* mutations, given the important role it may play in meningioma progression [7, 21].

## Results

We performed whole exome sequencing on four human meningioma cell lines (**Table 1**), including two from grade I meningiomas (HBL-52 and Ben-Men-1), one from an anaplastic meningioma (IOMM-Lee), and one from a meningioma resected in 1977 with unclear classification (CH157-MN), to characterize copy number alterations, mutations, and indels [22–25]. We also performed Sanger sequencing to validate identified mutations in known meningioma driver genes, along with previously identified hotspot mutations in these genes.

**Table 1:** Characteristics of the human meningioma cell lines.

Cell line	WHO grade	Location	Gender/age (y)	Reference
HBL-52	I	Optic canal	Female, 47	[25]
Ben-Men-1	I	Parietal falx	Female, 68	[24]
IOMM-Lee	III	Intraosseous	Male, 61	[22]
CH157-MN	unknown	unknown	Female, 41	[23]

WHO, World Health Organization.

### Copy number variation in human meningioma cell lines

**Fig 1:**
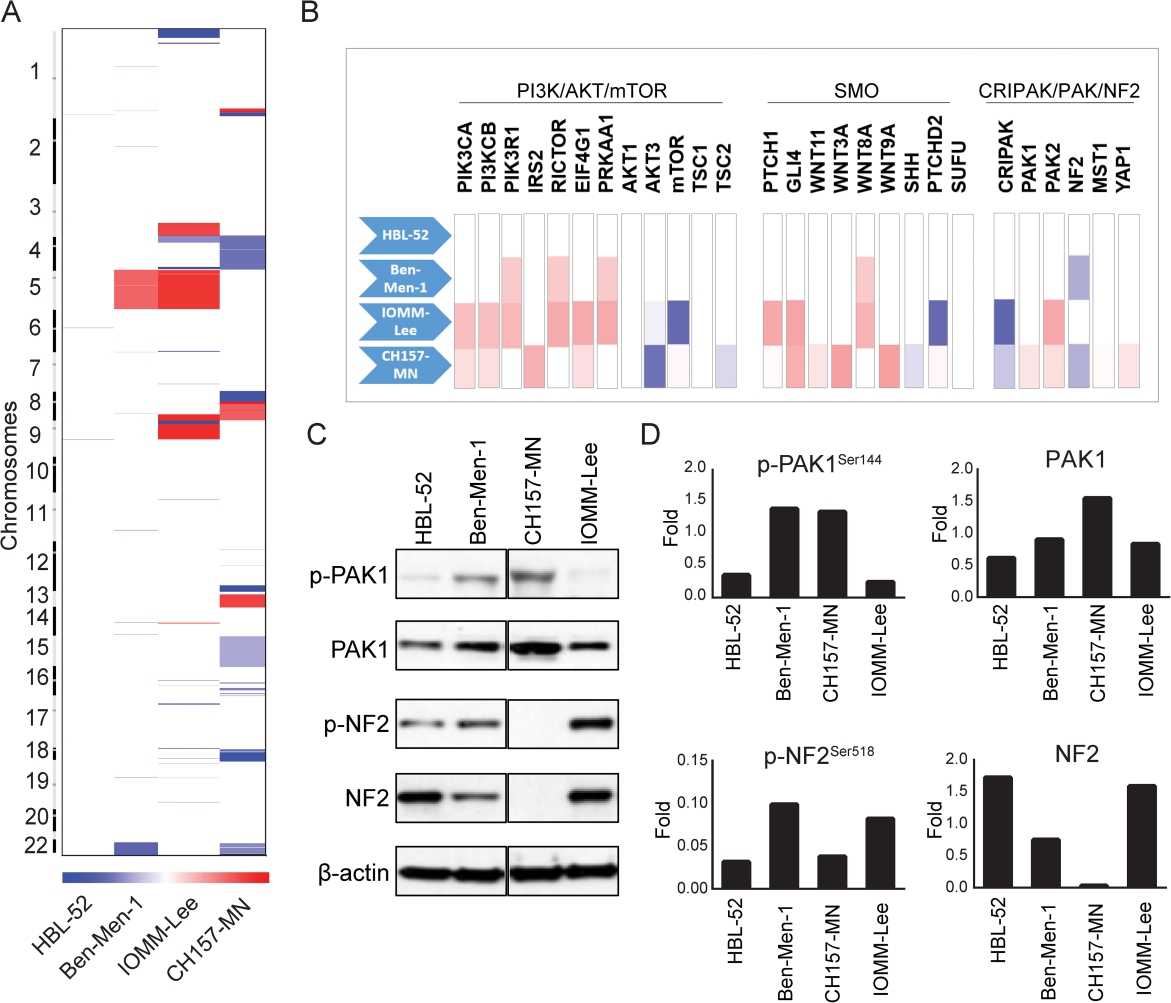
Somatic copy-number alterations in human meningioma cell lines. (A) Heatmap of chromosomal gains (red) and losses (blue) across all 22 chromosomes (y-axis) for four meningioma cell lines (x-axis). (B) Focal gains (red) or losses (blue) across gene members of three distinct signaling pathways (x-axis) for four meningioma cell lines (y-axis). (C) Western blot analysis of phosphorylated and un-phosphorylated PAK1 and NF2. (D) Quantification of western blot protein expression, p-PAK1 and p-NF2 were normalized to PAK1 and NF2, respectively. PAK1 and NF2 were normalized to loading control β-actin.

Arm-level chromosomal alterations are a hallmark of meningiomas, especially high-grade and angiomatous subtypes. CH157-MN harbored the greatest number of arm-level SCNAs of all four cell lines, consistent with the pattern observed in high-grade human meningiomas, while the grade I meningioma cell line HBL-52 harbored no broad copy number alterations (**Fig 1A**). We identified a number of arm-level gains and losses, including gain of 3p, 3q, 5p, 5q, 9p, and 13q, and loss of 4p, 4q, 8p, 8q, 15q, 18p,18q and 22q, across individual cell lines (**Fig 1A**). These losses span region including important oncogenes and tumor suppressors, including loss of cyclin dependent kinase inhibitor 2A (*CDKN2A)* on 9p21.3 in IOMM-Lee (**Table 2**).

**Table 2:** Copy number variations in human meningioma cell lines.

CELL LINE	CYTO BAND	CHROMOSOME	TYPE OF CNV	GENES AFFECTED
**CH157-MN**	1q42.11	Chr1:225142802–226187015	1044kb duplication	DNAH14,ENAH,EPHX1,LBR,LEFTY1,LEFTY2,SRP9,TMEM63A,SDE2
**CH157-MN**	1q42.11	Chr1:224606120–224922410	316kb duplication	CNIH3
**CH157-MN**	1q42.11	Chr1:226590082–226925161	335kb duplication	PARP1,c1orf95, ITPKB
**IOMM- Lee**	5p15	Chr5:801281–825368	24kb duplication	ZDHHC11
**IOMM- Lee**	9p21.3	Chr9:21971209–21974828	3.6kb deletion	CDKN2A
**CH157-MN**	12q24.22	Chr12:118504499–118509274	4.7kb deletion	VSIG10

We examined the copy number profile of specific genes within cardinal signaling pathways implicated in meningioma formation, including the phosphoinositide 3-kinase (PI3K) / mechanistic target of rapamycin (mTOR)/AKT and Hedgehog pathways, as well as cysteine-rich PAK1 inhibitor (CRIPAK) / p21 protein-activated kinase (PAK)/NF2 pathway (**Fig 1B**). We found that the PI3K/mTOR/AKT and SMO pathways were activated in 75% of human meningioma cell lines.

Loss of chromosome 22, which contains the known meningioma tumor suppressor *NF2*, was observed in Ben-Men-1 and CH157-MN. One negative regulator of Merlin, the protein product of *NF2*, is PAK1, which is in turn inhibited by CRIPAK [26, 27]. We observed loss of *CRIPAK* in CH157-MN and IOMM-Lee and gain of its possible downstream effectors, *PAK1/2* (**Fig 1B**). Western blot analyses of the meningioma cell lines corroborated high levels of protein expression of PAK1 and phospho-PAK1 and absence of NF2 in CH157-MN (**Fig 1C and 1D**).

### Mutation analysis in human meningioma cell lines

**Fig 2:**
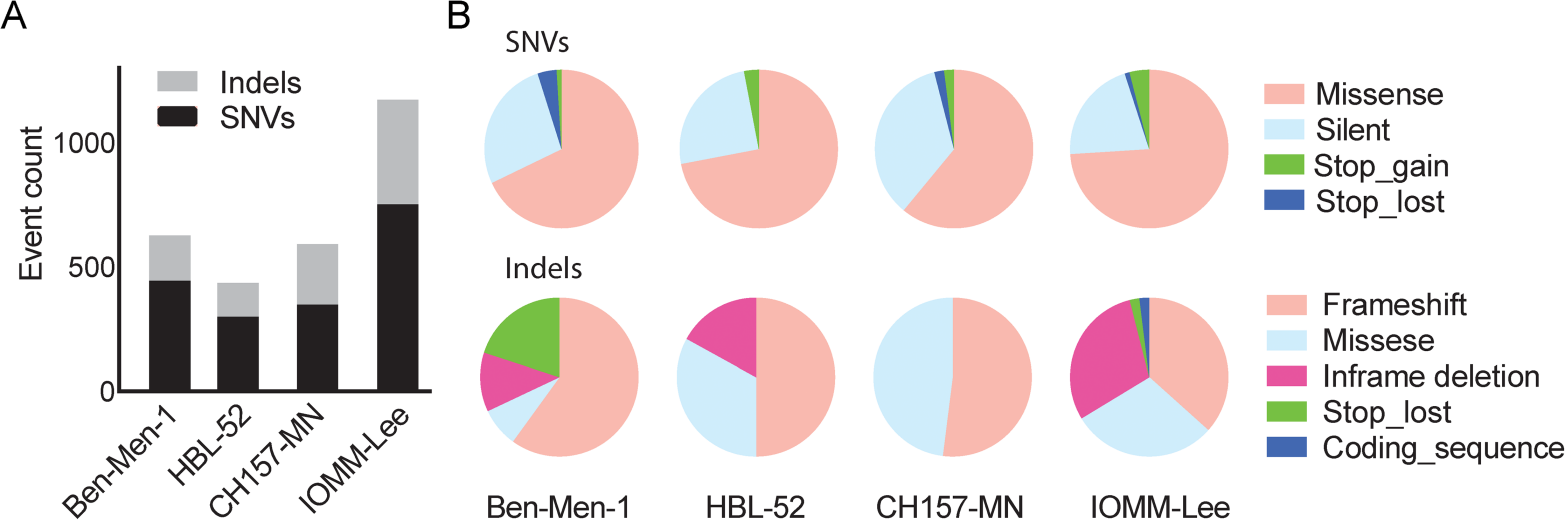
Mutations identified in human meningioma cell lines. (A) Number of mutations (y-axis) detected in each cell line (x-axis). (B) Distribution of coding consequences from SNV and Indel variants observed in meningioma cell lines.

**Fig 3:**
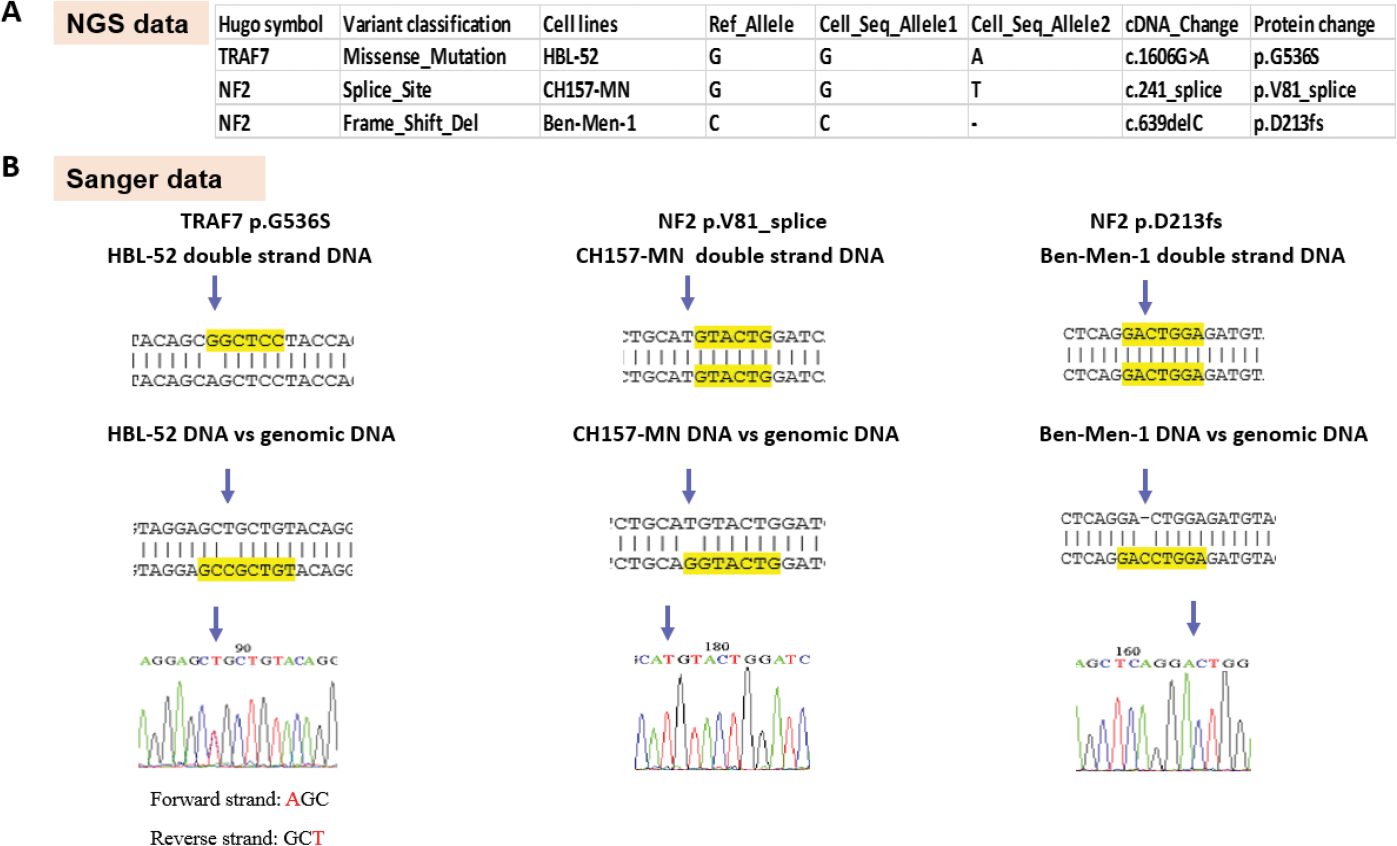
Validation of mutation calls human meningioma cell lines. (A) Table of putative meningioma driver-gene alterations identified from whole-exome sequencing. (B) Schematic demonstrating validation protocol for each mutation.

We identified a total of 2832 variants (mean 708) across the four cell lines, with a significantly higher number of mutations and indels in the anaplastic cell line IOMM-Lee compared to the other cell lines (**Fig 2A**). The most frequent variants were missense mutations and frameshift indels types across all cell lines (**Fig 2B**). Recurrent mutations and indels in *aquaporin 12B* (*AQP12B)*, *acetylserotonin O-methyltransferase-like (ASMTL)*, *CRIPAK*, and *SCO-Spondin* (*SSPO*) were identified across the cell lines (**Table 3**). We also identified mutations in known meningioma driver genes, including two mutations in *NF2* and a *TRAF7*^G536S^ missense mutation, which were confirmed by Sanger sequencing (**Fig 3**). We also performed Sanger sequencing to check for the presence of mutations previously reported in meningioma driver genes, including regions of *NF2*, *AKT1*, *TRAF7*, *KLF4*, *SMO*, and the *TERT* promoter. However, apart from the initial three mutations identified via NGS, we did not observe additional alterations, suggesting that our NGS coverage was sufficient for detection. *TERT* promoter mutation C228T (c.-124C>T), as assayed by focused sequencing, was observed in CH157-MN and IOMM-Lee (**Table 3**).

**Table 3:** Recurrent genetic variants in human meningioma cell lines.

SYMBOL	CHROMOSOME	VARIANT CLASSIFICATION	CDNA CHANGE	PROTEIN CHANGE	CELL LINES
**NBPF10**	chr1:144220807	Missense_Mutation	c.1793A>C	p.D598A	CH157-MN, Ben-Men-1
**SPDYE6**	chr7:101988983	Missense_Mutation	c.890C>T	p.P297L	IOMM-Lee, HBL-52, Ben-Men-1
**LOC399753**	chr10:49218451	Missense_Mutation	c.1688C>T	p.T563I	IOMM-Lee, Ben-Men-1
**DDX11L11**	chr12:92119	Missense_Mutation	c.191A>G	p.H64R	CH157-MN, HBL-52
**TBC1D3P2**	chr17:60345508	Nonstop_Mutation	c.760T>C	p.*254Q	CH157-MN, IOMM-Lee, HBL-52
**SIGLEC16**	chr19:50474960	Missense_Mutation	c.14C>G	p.A5G	HBL-52, Ben-Men-1
**KIR2DL2**	chr19:35127	Missense_Mutation	c.796C>T	p.R266C	CH157-MN, Ben-Men-1
**KIR2DL2**	chr19:39293	Missense_Mutation	c.52C>A	p.P18T	CH157-MN, Ben-Men-1
**KIR2DL2**	chr19:118836	Missense_Mutation	c.313A>C	p.T105P	HBL-52, Ben-Men-1
**KIR2DL2**	chr19:118856	Missense_Mutation	c.333G>T	p.L111F	HBL-52, Ben-Men-1
**AQP12B**	chr2:241621800–241621800	Frame_Shift_Del	c.455delG	p.S152fs	IOMM-Lee, HBL-52
**CRIPAK**	chr4:1388375–1388376	Frame_Shift_Ins	c.76_77insCA	p.S26fs	CH157-MN, Ben-Men-1
**SSPO**	chr7:149503917–149503920	Splice_Site	c.8736_splice	p.G2912_splice	IOMM-Lee, CH157-MN, HBL-52
**LOC399753**	chr10:49218408–49218408	Frame_Shift_Del	c.1731delC	p.N577fs	CH157-MN, HBL-52
**ASMTL**	chrx:1522164–1522164	Frame_Shift_Del	c.1864delT	p.*622fs	IOMM-Lee, CH157-MN, HBL-52
**TERT**	chr5:1295228	Missense_Mutation	c.-124C>T		IOMM-Lee, CH157-MN

### Chromosome rearrangements in human meningioma cell lines

**Fig 4:**
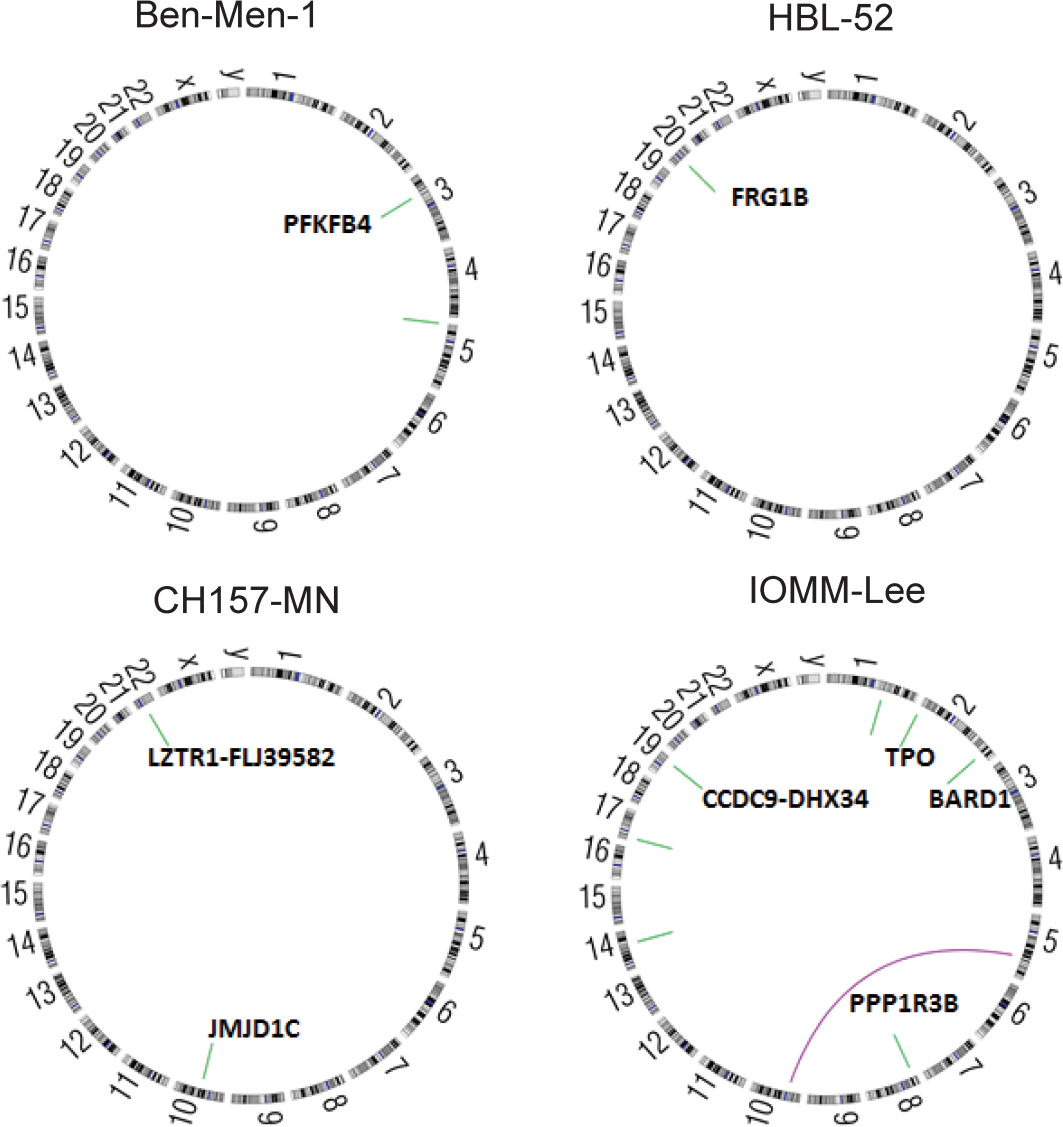
Rearrangements detected in human meningioma cell lines. Circos plots showing intra-chromosomal (green) and inter-chromosomal (purple) rearrangements between regions of the genome.

Although whole-exome sequencing is underpowered to detect chromosomal rearrangements, we identified 14 rearrangements across the four cell lines (**S2 Table**). Of note, in CH157-MN harbored an intrachromosome translocation of *jumonji domain containing 1C* (*JMJD1C)* on chromosome 10, resulting in deletion of this gene, and a fusion of *leucine zipper like transcription regulator 1* (*LZTR1)* and *FLJ39582* on chromosome 22 (**Fig 4**).

## Discussion

*In vitro* models have played important roles in furthering our understanding of cancer biology and treatment paradigms across a range of tumor types. As our understanding has increased of the impact that molecular classification has on patient response to treatment, it has become crucial to determine whether model systems accurately recapitulate the disease of interest. We performed the first whole-exome sequencing of meningioma cell lines to determine their applicability to the study of meningioma biology.

Recent work has demonstrated that there exist two broad classes of meningiomas based on their mutational profile: those driven by *NF2* inactivation, and those with non-*NF2* driver gene alterations, such as mTOR and Hedgehog pathway alterations [2–5]. Such differences in tumorigenesis may significantly impact the consequent signaling pathways. We show that two commonly used cell lines, CH157-MN and Ben-Men-1, harbor deleterious *NF2* mutations as well as chromosome 22 loss. Furthermore, interrogation of signaling partners in the NF2 cascade revealed high levels of PAK1, an upstream oncogene that inactivates NF2 in cancer cells through phosphorylation [28]. These results suggest that CH157-MN and Ben-Men-1 could be well suited to study *NF2* signaling in meningioma formation. We identified a canonical mutation in *TRAF7* in an additional cell line, HBL-52, which may be more amenable to studies examining the signaling pathways important in non-*NF2* driven tumors.

We detected no mutations in either *AKT1*, *SMO*, or *PIK3CA* in these four profiled cell lines. These well-established driver alterations result in activation of pathways that are amenable to pharmacologic inhibition [2–4], with clinical trials testing inhibitors against these drivers underway. New *in vitro* models that replicate these oncogenic pathways are needed to better understand the unique molecular pathways influenced by these non-NF2 mutations as well as to investigate mechanisms for acquired resistance with application of targeted inhibitors.

Genomic instability is one of the key differentiators between grade I and grade II-III meningiomas [29]. Loss of chromosome 22 is the most common arm-level alteration across all meningiomas (40–60% in grade I, 75% in grade II-III), along with recurrent loss of chromosomes 1p, 6q, 10q, 14q, and 18q, and gain of 1q, 9q, 12q, 15q, 17q and 20q in high-grade tumors. We found a higher level of genomic disruption in the two cell lines, IOMM-Lee and CH157-MN, consistent with the original meningioma being of a high-grade nature. Of note, the pattern of copy number alterations in these cell lines differs from the most commonly observed altered chromosomal losses and gains in human meningioma, which may reflect genomic changes over time during *in vitro* passages or be specific to the original tumors from which the lines were derived.

We observed a number of potentially oncogenic focal amplifications and deletions. Loss of chromosome 9p21.3, which harbors the critical tumor suppressor *CDKN2A*, was observed in IOMM-Lee, a cell line derived from an anaplastic meningioma. CH157-MN had gain of chromosome 1q, along with a series of focal gene changes on 1q42.11. Chromosome 5 was amplified in both Ben-Men-1 and IOMM-Lee, with corresponding increase in copy number of genes associated with vascular development and proliferation, such as platelet derived growth factor subunit b *(PDGFβ)*, fibroblast growth factor receptor 4 (*FGFR4)*, and fibroblast growth factor receptor 10 (*FGF10)*. We also detected amplification of both enabled homolog (*ENAH)* and poly [ADP-ribose] polymerase 1 (*PARP1)* in CH157-MN, although these genes have not previously been implicated in meningiomas tumorigenesis [30, 31].

We previously demonstrated that meningiomas of all grades present with complex rearrangement profiles, including frequent observation of complex events such as chromothripsis and chromoplexy [2]. While whole-exome sequencing is not powered to identify most rearrangements, we were able to detect a number of intrachromosomal rearrangements and one interchromosomal rearrangement, involving genes that have previously been implicated in tumorigenesis, including BRCA1 Associated RING Domain 1 (*BARD1)*, *JMJD1C* [32], and *LZTR1*. However, none were recurrently rearranged in our cohort, nor have they previously been shown to be recurrently mutated in meningioma. Further investigation of whole-genome sequenced meningioma will be necessary to identify oncogenic rearrangements.

In summary, this study identified the aberrance of chromosome structure, focal gene changes, and somatic single nucleotide variants in human meningioma cell lines. The alterations of these genes might be involved in meningioma progression by affecting cell motility, cytokinesis, chromatin and epigenomic regulation, immune response, malignant transformation, or metabolism. Due to the limited meningioma cell lines currently available, much work remains to complete the mutational catalog of meningiomas to connect recurrent genomic alterations to altered pathways and acquired cellular vulnerabilities, and their correlation with disease stratification and prognosis. Moreover, future insights into the molecular mechanisms of these genetic drivers in meningioma development might inform our understanding of genome influence on meningioma evolution and therapy.

## Supporting information

S1 Table Primers used for validation of variants. (DOCX)

S2 Table Rearrangement analysis of human meningioma cell lines. (DOCX)

## Abbreviations

AKT1: v-akt murine thymoma viral oncogene homolog 1
AKT3: v-akt murine thymoma viral oncogene homolog 3
AQP12B: aquaporin 12B
ASMTL: acetylserotonin O-Methyltransferase-Like
BAP1: BRCA1 Associated Protein 1
BARD1: BRCA1 Associated RING Domain 1
CDKN2A: cyclin dependent kinase inhibitor 2A
CRIPAK: cysteine-rich PAK1 inhibitor
ENAH: enabled homolog (Drosophila)
FGF10: fibroblast growth factor receptor 10
FGF4: fibroblast growth factor receptor 4
GATK: genome analysis toolkit
IGV: integrative genomics viewer
KLF4: krupplelike factor 4
JMJD1C: jumonji domain containing 1C
LZTR1: leucine zipper like transcription regulator 1
mTOR: mechanistic target of rapamycin
NF2: neurofibromin 2
PAK1: p21 protein-activated kinase 1
PARP1: poly [ADP-ribose] polymerase 1
PDGFb: platelet derived growth factor subunit b
PI3K: phosphoinositide 3-kinase
PIK3CA: phosphoinositide-3-kinase, catalytic, alpha polypeptide
POLR2A: RNA polymerase II subunit A
SCNAs: somatic copy-number alterations
SMARCB1: SWI/SNF related, matrix associated, actin dependent regulator of chromatin, subfamily b, me SSPO, SCO-Spondin
SUFU: suppressor of fused, drosophila, homolog of
TERT: telomerase reverse transcriptase
TRAF7: TNF receptor-associated factor 7
VEP: variant effect predictor
WES: Whole exome sequencing
