# Supplementary material for: Genomic profile of human meningioma cell lines
Source: PLoS One. 2017 May 26;12(5):e0178322. doi: 10.1371/journal.pone.0178322 (PMC5446134; doi:10.1371/journal.pone.0178322)
Supplement: S1 Table — (DOCX) [file pone.0178322.s001.docx]

**S1Table. Primers used for validation of variants.**

*** TERT promoter C228T, C250T**
