# Supplementary material for: Genomic profile of human meningioma cell lines
Source: PLoS One. 2017 May 26;12(5):e0178322. doi: 10.1371/journal.pone.0178322 (PMC5446134; doi:10.1371/journal.pone.0178322)
Supplement: S2 Table — (DOCX) [file pone.0178322.s002.docx]

**S2 Table. Rearrangement analysis of human meningioma cell lines**

| **chr1** | **pos1** | **chr2** | **pos2** | **site1** | **site2** | **class: fusion details** | **cell line** |
| --- | --- | --- | --- | --- | --- | --- | --- |
| **chr1** | 157967402 | chr1 | 158047671 | Intron of KIRREL(+): 4Kb after exon 1 | Intron of KIRREL(+): 64bp before exon 3 | Deletion of 1 exon: in frame | IOMM-Lee |
| **chr2** | 1459935 | chr2 | 1469302 | Exon 7 of TPO(+) | Intron of TPO(+): 9Kb after exon 7 | Deletion within transcript: mid-exon | IOMM-Lee |
| **chr2** | 215657049 | chr2 | 215667142 | Exon 3 of BARD1(-) | Intron of BARD1(-): 5Kb before exon 2 | Deletion within transcript: mid-exon | IOMM-Lee |
| **chr3** | 48555851 | chr3 | 48559362 | 3'-UTR of PFKFB4(-): 1Kb after coding stop | Intron of PFKFB4(-): 40bp after exon 13 | Deletion | Ben-Men-1 |
| **chr5** | 126206302 | chr10 | 33168466 | 3'-UTR of MARCH3(-): 34bp after coding stop | 3'-UTR of C10orf68(+): 3Kb after coding stop | inter_chr | IOMM-Lee |
| **chr8** | 8998838 | chr8 | 9007539 | Exon 2 of PPP1R3B(-) | 5'-UTR of PPP1R3B(-): 8Kb before coding start | Deletion within transcript: mid-exon | IOMM-Lee |
| **chr10** | 64950653 | chr10 | 64966605 | Intron of JMJD1C(-): 2bp after exon 17 | Exon 10 of JMJD1C(-) | Deletion within transcript: mid-exon | CH157-MN |
| **chr14** | 67629783 | chr14 | 68244907 | Intron of GPHN(+): 2Kb before exon 20 | Exon 24 of ZFYVE26(-) | tandem_dup | IOMM-Lee |
| **chr17** | 7143277 | chr17 | 7481247 | Promoter of PHF23(-): 458bp from tx start | Intron of EIF4A1(+): 32bp after exon 9 | Deletion | IOMM-Lee |
| **chr19** | 47761885 | chr19 | 47849914 | Exon 3 of CCDC9(+) | Promoter of DHX34(+): 3Kb from tx start | Inversion: antisense fusion | IOMM-Lee |
| **chr20** | 29623254 | chr20 | 29625870 | 5'-UTR of FRG1B(+): 11Kb before coding start | 5'-UTR of FRG1B(+): 8Kb before coding start | Deletion within transcript | HBL-52 |
| **chr22** | 21341936 | chr22 | 21358092 | Intron of LZTR1(+): 70bp after exon 4 | 5'-UTR of FLJ39582(+): 7Kb before coding start | Deletion: transcript fusion (LZTR1-FLJ39582) | CH157-MN |
